# A method for discovering and inferring appropriate eligibility criteria in clinical trial protocols without labeled data

**DOI:** 10.1186/1472-6947-13-S1-S6

**Published:** 2013-04-05

**Authors:** Angelo Restificar, Ioannis Korkontzelos, Sophia Ananiadou

**Affiliations:** 1 National Center for Text Mining & School of Computer Science, The University of Manchester, Manchester, M1 7DN, UK

## Abstract

**Background:**

We consider the user task of designing clinical trial protocols and propose a method that discovers and outputs the most appropriate eligibility criteria from a potentially huge set of candidates. Each document *d *in our collection *D *is a clinical trial protocol which itself contains a set of eligibility criteria. Given a small set of sample documents$D^{\prime },\;\;|D^{\prime }|\;\;\ll \;\;|D|$, a user has initially identified as relevant *e.g.*, via a user query interface, our scoring method automatically suggests eligibility criteria from *D, D *⊃ *D'*, by ranking them according to how appropriate they are to the clinical trial protocol currently being designed. The appropriateness is measured by the degree to which they are consistent with the user-supplied sample documents *D'*.

**Method:**

We propose a novel three-step method called LDALR which views documents as a mixture of latent topics. First, we infer the latent topics in the sample documents using Latent Dirichlet Allocation (LDA). Next, we use logistic regression models to compute the probability that a given candidate criterion belongs to a particular topic. Lastly, we score each criterion by computing its expected value, the probability-weighted sum of the topic proportions inferred from the set of sample documents. Intuitively, the greater the probability that a candidate criterion belongs to the topics that are dominant in the samples, the higher its expected value or score.

**Results:**

Our experiments have shown that LDALR is 8 and 9 times better (resp., for inclusion and exclusion criteria) than randomly choosing from a set of candidates obtained from relevant documents. In user simulation experiments using LDALR, we were able to automatically construct eligibility criteria that are on the average 75% and 70% (resp., for inclusion and exclusion criteria) similar to the correct eligibility criteria.

**Conclusions:**

We have proposed LDALR, a practical method for discovering and inferring appropriate eligibility criteria in clinical trial protocols *without *labeled data. Results from our experiments suggest that LDALR models can be used to effectively find appropriate eligibility criteria from a large repository of clinical trial protocols.

## Background

One of the important considerations that a clinical researcher makes when putting together a clinical trial protocol is deciding who should and should not participate in the planned clinical trial based on a list of criteria. Such information is usually explicitly stated in the document as inclusion and exclusion criteria. The inclusion and exclusion criteria play an important role in that they specify the characteristics of the sample population under study and therefore the results and conclusions of the clinical trial is valid only to the extent that this sample group represents the entire population for which a potential treatment or drug is targeted. To help clinical researchers in this task, we have developed a technique called LDALR [1], that automatically discovers and infers appropriate eligibility criteria, *i.e.*, criteria not necessarily contained in the documents initially identified by the user as relevant. This technique has been implemented and is part of a larger interactive system [2] that uses text mining tools.

To assess and identify the appropriate criteria our approach leverages a machine learning technique where supervised models are trained by using as labels the output of an unsupervised machine learning method. In particular, we train regularized logistic regression models [3,4] on data with latent topics as labels previously identified by Latent Dirichlet Allocation [5]. We score each criterion by computing its expected value, the probability-weighted sum of the topic proportions inferred from the set of sample documents, initially supplied by the user. Our technique can be described as using topic proportions as a form of 'signal signature' to characterize both user-supplied documents and criteria candidates. Intuitively, the greater the probability that a candidate criterion belongs to the topics that are dominant in the samples, the higher its expected value or score.

As part of our plan to conduct user study to more effectively assess and evaluate our technique, we have also developed an eligibility criteria comparison tool. The tool is accessible online and is intended to facilitate the elicitation of user-provided eligibility criteria rank ordering by allowing a domain expert or clinician to easily view results from different methods. This paper discusses in detail the aforementioned technique, LDALR, and the eligibility criteria comparison tool.

## Related work

An earlier system, ASCOT [6], was designed with a goal similar to the work we present in this paper. ASCOT is a search system focusing on clinical trial protocols, which aims to aid medical practitioners and clinical researchers in developing new clinical trial protocols. The component of ASCOT that is relevant to the work described in this paper is its eligibility criteria recommendation engine. LDALR differs from ASCOT in at least two major ways: (a) LDALR is designed to discover relevant eligibility criteria even if these criteria are not in the set of documents selected by the user, and (b) it uses latent topics inferred from the documents selected by the user as relevant, to determine the importance of an eligibility criterion. In contrast, ASCOT does not retrieve related criteria from protocols that are not selected by the user. It only scores the set of criteria from the collection of documents selected by the user, and presents them in order of decreasing importance. To determine the importance of an eligibility criterion, ASCOT uses the frequency of biomedical concepts which are computed by applying METAMAP [7] on each eligibility criterion text. Eligibility criteria that have higher UMLS concept frequency are given higher weight and are considered more important.

In the clinical question-answering (QA) domain, the work that is closest to what we are doing especially in terms of architecture is that of Demner-Fushman and Lin [8]. While Demner-Fushman and Lin focused on Medline abstracts their QA system architecture is general enough that it is applicable to our domain. The task of finding the appropriate eligibility criteria can also be framed as a specific instance of a clinical QA problem. In particular, one can ask 'What is the most appropriate set of eligibility criteria?', *i.e.*, for the particular clinical trial protocol the user is putting together. Indeed, in terms of Demner-Fushman and Lin's architecture the output of the work discussed here is analogous to what their Semantic Matcher and Answer Generator modules produce. One of the main differences between Demner-Fushman and Lin's work and ours is that that our parameters and models are automatically learned from data while the weights they assign to compute their numeric scores are 'ad hoc and are based on intuition' [8], *i.e.*, subjectively set by a domain expert. In terms of scalability and maintenance cost, our approach would have more advantage since it is fully automatic and requires less effort to maintain and update. Another difference between their work and what is presented here is that while their system attempts to answer more general questions the task of our system is focused on just answering one type of question, *i.e.*, on finding the most appropriate set of eligibility criteria. Our method can potentially be applied to answer more general questions, for instance, instead of taking the cue from a small set of documents initially supplied by the user, data taken from the question itself can be used to score candidate answers.

Patel and Cimino [9] describe a tool that would allow search of eligible patients in a database through a formal query representation of an eligibility criterion. In their work, eligibility criteria are extracted from an XML document and UMLS concepts are identified via MetaMap (MMTx). Irrelevant concepts are filtered out by semantic types and the relevant ones are mapped to a target terminology used to encode the database. Another work that leverages domain-specific knowledge like UMLS is that of Korkontzelos et al. [6] (ASCOT). The recommendation engine used in ASCOT uses UMLS concept counts instead of the machine learning models discussed in this paper. The LDALR technique we have formulated in this work, however, is general enough so that enhancing our data with UMLS concepts, *e.g.*, in a manner similar to how we have used PICO or by transforming raw data into some intermediate form using UMLS SNOMED CT terms and ontology, can easily be done. This can potentially reduce the number of features and could lead to better recommendations. In contrast to the approach taken in ASCOT, LDALR does not totally depend on domain-specific knowledge such as UMLS, hence it is more flexible and can be applied to other domains, and is readily able to make recommendations with or without the explicit use of domain-specific knowledge. In addition, the availability of open-source parallel and distributed computing environments like Apache Hadoop (hadoop.apache.org) which can run on commodity computers make tools that can exploit parallel computation, like LDALR, very practical solutions to solving complex problems.

The aim of Bruijn et al.'s work [10] is to extract from full text Randomized Clinical Trials (RCT) journals over 20 information elements like eligibility criteria, the name of experimental and control treatments, intervention parameters like dosage, frequency, duration, etc. The authors use a text classifier (SVM) to identify the sentence(s) that most likely contains the target information element then proceeds to extract that element via a regular expression match. The classifiers were trained on 78 annotated articles and tested on a hold-out of 10 articles. According to the authors, their method can locate the correct snippet in 75% of the cases, where 35% obtained perfect precision and recall. When tested on unseen articles, the method extracted the eligibility criteria with a precision of 0.69 and a recall of 0.54. Recent results from a system called ExaCT [11], based on [10] but with refined core algorithms and pattern rules, put the precision and recall numbers at 0.78 and 0.78, respectively. As part of our future work, we will be using similar techniques used in [10,11] to extract eligibility criteria and other relevant information in documents that are not XML-tagged.

## Methods

Given a potentially huge set of candidate criteria, our goal is to help users choose the most appropriate criteria from among the candidates. In this work, we assume that the candidate criteria exist in some document(s) in a given repository, either separately from different documents or together in the same document, and our technical objective is to rank these candidates according to their appropriateness to the task at hand. As mentioned previously, such task would be to put together a clinical trial protocol about some particular disease, treatment, or drug.

To provide a concrete idea of our problem, the following is a snippet of a clinical trial protocol, downloaded from the clinical trials website http://www.clinicaltrials.gov, and modified for presentation as an example. It shows the title, the objective of the clinical trial as well as the inclusion and exclusion criteria.

Title: Antioxidant Systems and Age-Related Macular Degeneration Objective:

The objective of this study is to determine whether the antioxidant supplements used in AREDS shifted the plasma pool of the AREDS subjects to a more reduced state.

The AREDS subjects were randomly assigned to one of four treatment groups:

1. antioxidants (500mg Vitamin C, 4000IU Vitamin E, 15mg beta carotene)

2. zinc (80mg zinc oxide, 2mg cupric oxide)

3. antioxidants plus zinc;

4. placebo.

...

Inclusion Criteria:

* Age 55-80

* Participants with Intermediate or Advanced AMD

* participants with no ocular signs of AMD

* Willing to give written informed consent, make the required study visits, and follow instructions

* Any race and either sex

Exclusion Criteria:

* Current history of a medical condition that would preclude scheduled study visits or completion of the study (e.g., unstable cardiovascular disease, unstable pulmonary disease, chronic hepatitis, or AIDS).

* Intraocular surgery in study eye (eye to be treated) within 60 days prior to enrollment

* Presence of a scleral buckle in the study eye

...

Clearly, as the snippet above shows, the solution to the problem of automatically identifying the appropriate eligibility criteria is not an easy task. There are many possible starting points from which to address this problem ranging from knowledge-based approaches [8], use of techniques involving natural language processing [9,12], information extraction methods [10,12,13], to the use machine learning approaches [5,14,15].

In this section, we describe an approach using machine learning techniques, in particular the use of topic models and probability models. One practical advantage in using probability models is that assuming availability of sufficient training data, probability models are fairly easy to build and update. In contrast to knowledge-based approaches in which domain knowledge from experts are used, typically no manual tuning is required in machine learning approaches because general 'rules' are automatically inferred or discovered from observations.

For our particular problem, however, it is not quite obvious how to leverage probability models since we do not have data labeled as appropriate or inappropriate eligibility criteria. This means no supervised machine learning method can be directly used to solve the modeling problem. Even if one wants to construct such a training data, *e.g.*, with appropriate (+1) and inappropriate (−1) labels, it would be infeasible to construct one that can be used for *any *given search string associated with the task of putting together clinical trial protocols.

Our approach to addressing the unavailability of training data is to combine an unsupervised method and a supervised method to eventually construct probability models. The intuition is to identify a set of random variable(s) that would allow us to specify conditions on the probability distribution. Topics can naturally be these random variables and topics associated with a candidate criterion can be used to specify conditions on a probability distribution. For instance, the probability that a candidate criterion is a good solution is directly linked to whether that candidate criterion belongs to the same set of topics a user is interested in, *i.e.*, conditional upon the same set of topics.

Topic modeling [5,15], an unsupervised machine learning approach, is a technique that can be used to infer what these topics are. Once these topics have been identified, it is possible to build probability models or classifiers using a one-versus-rest approach as a way of assigning training labels. Given training data, we can use topic models to identify in a particular set of documents these topics. These topics are themselves expressed as a group of words that belong to particular documents. We shall refer to this supervised training technique as *anchored training*, where topics predicted using an unsupervised learning model are subsequently used as pseudo-labels to build models using a supervised learning method. In a sense, the training 'labels' for the supervised learning method are based or 'anchored' on latent topics which were inferred without manual labeling. It is important to point out that although topic models output clusters of documents according to particular topics, these topics are really pseudo-labels in the sense that documents vary in their levels of membership to a particular cluster. Unlike standard labels used in supervised learning methods which clearly signify a training instance to be of one label and not the other, there is some amount of noise associated with our pseudo-labels. A document (or in our case, a training instance) could be a member of more than one cluster or topic in varying levels of proportion so it is not readily apparent that these pseudo-labels are useful without further refinement.

The first step in building a model through anchored training is to get a rough idea about which subsets of the data are good training candidates for a particular pseudo-label. This step is achieved through an unsupervised learning method. Our goal in using an unsupervised method is to get an approximate grouping of data. This approximate grouping via latent topics (pseudo-labels) can then be used as a guide to tease out and determine the features that have real significant predictive or discriminatory power. We then apply a model refinement step using a supervised learning method. Optionally, this model refinement step can be done after a dimensionality reduction phase. In the second step, the output is a refined model because important attributes or features that are useful are teased out and determined, *e.g.*, via the attributes' corresponding learned weights, during the model fitting process. Thus, anchored training provides a way to build supervised models without the high cost associated with manually labeling a set of training instances. More importantly, models could potentially be built even in the case where no labeled training data is available. The training method is outlined in Algorithm 1 where line (2) can be replaced with any method that outputs topic weights

**Algorithm 1: **Anchored Training

Let *W ***= {***w_i_***} **denote topic proportions, and *D *collection of documents *d*. Let *P *= {*p_i_*}, *i *= 1 ... *n *be a set of trained logistic regression models *p_i_*, and *LDA_n _*be a trained Latent Dirichlet Allocation (LDA) model, where *n *is the number of topics.

**Input: **set of documents *D*, thresholds *κ*_1_, *κ*_2_

**Output: **set of probability models *P*

**Anchor**(*D*)

(1) *P ***← **∅

(2) *W *← *LDA_n_*(*D*)

(3) **foreach ***i *= 1 ... n

(4)    *L_i _*← {*d *∈ *D*| *w_i_*(*d*) > κ_1_}

(5)    *L_−i _*← {*d *∈ *D*| *w_i_*(*d*) ≤ κ_2_}

(6)    *p_i _*← train(*L_i_, L_−i_*)

(7)    *P *← *P *∪ {*p_i_*}

(8) **return ***P*

or proportions and where line (6) can be replaced with any supervised method that uses the labels *L_i_, i *= 1 ... *n*.

In the work reported here, we used Latent Dirichlet Allocation (LDA) [5] as the unsupervised learning method for anchored training. Given a document, LDA outputs the topic proportions that document has. With a sufficiently large collection of documents, it is then easy to find documents that have a topic *t *in their mixture and those that do not have a topic *t*. Alternatively, threshold values as suggested in lines (4)-(5) in Algorithm 1, can be used. Clearly, one can now use this dichotomized data as data for either training classifiers or building probability models. In our case, we trained probability models from such data using logistic regression [4].

To help users put together the inclusion and exclusion criteria, we assume as an input to our method a small sample of clinical trial protocols identified by the user as relevant to the task at hand. Depending on the user interface, the sample set could be used as an initial seed to find other relevant documents. Such sample documents can, for instance, be a set resulting from a search query over a large repository by using a series of search strings. Note that as in the sample snippet above, important keywords that users normally use as part of the search string may or may not appear as words in the eligibility criteria section of the clinical trial protocol.

As mentioned above, the specific implementation of our approach uses Latent Dirichlet Allocation (LDA) and Logistic Regression (LR). We shall henceforth refer to our method as the LDALR method. Algorithm 2 presents the general steps of the LDALR method. In line (1), we use an LDA trained model to infer the latent topics from a given set of documents *D*. In this work, each *d *∈ *D *is a clinical trial protocol which in itself contains a set of criteria *S*. Each criterion *s_j _*∈ *S *can either be an inclusion criterion or an exclusion criterion, but not both. Then in line (2), we invoke the function GETSCORE (Algorithm 3) that executes two additional general steps. In GETSCORE, line (4) performs two steps. First, it uses the logistic regression models *p_i _*to compute the probability that a candidate criterion is of

**Algorithm 2: **LDALR Method

Let *W ***= **{*w_i_*} denote topic proportions, *s_j _*∈ *S *denote a criterion, and *S ***= **{*s_j _***| ***s_j _*∈ *d, d *∈ *D*}. Let *P ***= **{*p_i_*}, *i ***= **1 ... *n *be the set of trained logistic regression models *p_i_*, and *LDA_n _*be a trained Latent Dirichlet Allocation (LDA) model, where *n *is the number of topics.

**Input: **set of documents *D*, trained logistic regression models *P*, trained LDA model *LDA_n _***Output: **set of ordered criteria *S*^★^.

**LDALR**(*D, P, LDA_n_*)

(1) *W *← *LDA_n_*(*D*)

(2) *S*^★ ^← GETSCORE(*S, W, P*)

(3) **return ***S*^★^

topic *i*. This is done for all topics *i = *1 *... n*. Then, it computes the expected value *σ***(***s***) **of a candidate criterion by taking the sum of the probability-weighted normalized topic proportions $\tilde{w}$. GETSCORE returns a set of criteria sorted in descending order by each candidate criterion's expected value. Intuitively, any set of candidate criteria whose topic proportions are more dominant in the samples, will have higher expected values or scores compared to those whose topics are not significantly represented in the samples.

Each *d *∈ *D *has a topic proportion as computed in line (1) of Algorithm 2. In Algorithm 3, we simply normalize the topic proportions *w_i _*so that for a given $D,\;\overset{n}{\underset{i=1}{\sum }}w_{i}=1$. If we let *w_id _*= *w_i_*(*d*), denote the topic proportion of topic *i *in *d*th document, for *d *∈ *D*, then the normalized weights ${\tilde{w}}_{i}$ are simply computed as:

(1) $${\tilde{w}}_{i}=\frac{\sum_{d=1}^{\left|D\right|}w_{id}}{\sum_{i=1}^{n}\sum_{d=1}^{\left|D\right|}w_{id}}$$

The normalize function in line (2) of the Scoring Function is computed using Eq. (1).

The solutions given by LDALR have some interesting characteristics.

**Proposition 1 ***Let S be a set of criteria and H ***= {***h_j_***} ***be a sufficiently large set of randomly drawn target criterion h_j_, where H and S are possibly disjoint, and that the topics in H and S are drawn from the same distribution*. *Furthermore, let ϕ be any similarity function such that ϕ *(*x*_1_, *x*_2_) → [0, 1], *for x*_1_, *x*_2 _∈ *H *∪ *S and*

(2) $$s^{⊤}=\underset{s\in S}{\text{argmax}\phi (s,{\text{h}}_{j})}$$

(3) $$s^{*}=\underset{s\in S}{\text{argmax}\sigma (s)}$$

*where σ***(*s*) ***is the scoring function in Algorithm 3, then*

(4) $$\frac{\phi \left(s^{⊤},h_{j}\right)-\phi \left(s^{⊤},s^{*}\right)}{\left|H\right|}\;\;>\;\;\varepsilon$$

(5) $$\phi \left(s^{⊤},s^{*}\right)-\phi \left(s^{⊤},random\left(s\right)\right)\;\;>\;\;0$$

*for some ε ***>**0*, s *∈ *S, h_j _*∈ *H*

**Algorithm 3: **Scoring Function

Let *σ *denote the score of a criterion *s *∈ *S *and let *T *(***s***) denote the topic of *s*.

**Input: **set of criteria *S*, topic proportions *W ***= **{*w_i_*}, logistic regression models *P ***= **{*pi*}, for *i *= 1 ... *n*

**Output: **set of ordered criteria *S*^★^.

GETSCORE(*S, W, P *)

(1) *S***^★ ^← **∅

(2) $\tilde{W}\leftarrow \text{normalize}\left(W\right)$

(3) **foreach ***s *∈ *S*

(4)    $\sigma \left(s\right)\leftarrow \overset{n}{\underset{i=1}{\sum }}{\tilde{w}}_{i}p_{i}\left(T\left(s\right)=i\right)$

(5)    *S*^★ ^← *S*^★ ^∪ {〈*s, σ*(*s*)〉}

(6) **return **sort(*S*^★^)

Eqs. (4) and (5) describe some characteristics regarding average similarity, as a result of using LDALR. Eq. (4) simply says that the average similarity between what LDALR recommends as a solution and the optimal solution can not be more than the average maximum similarity between the target and any candidate. On the other hand, Eq. (5) establishes the lower bound on solutions that LDALR recommends *i.e.*, on the average, LDALR's solution can not be worse than a randomly drawn solution.

## Results and discussion

In this section, we discuss our implementation and the results of the experiments we have undertaken, as well as provide details of the eligibility criteria comparison tool. LDALR was implemented in Python using open-source toolkits to build LDA and LR models. To build our topic models, we used the Mallet toolkit [16] and to train our logistic regression models we used the LIBLINEAR toolkit [3,4]. In practice, all required values except the topic proportions associated with the user-selected sample documents, which can only be computed after the user has identified a small set of relevant documents, can essentially be pre-computed offline. The pre-computed values can then be simply stored in a look-up table for fast retrieval, *i.e.*, with constant, *O*(1), time complexity.

## Data and features

The data we used both for training and evaluation were downloaded from the clinical trials website, http://www.clinicaltrials.gov, which collects clinical trials data conducted in the United States and around the world. In order to measure the effectiveness of LDALR, we divided our dataset into two sets: a dataset for training (*L*) and a dataset for testing (*Q*). The data collection we used for our experiment has a total of 44,203 documents, in which 90% (39,782) were used for training and the remaining 10% (4,421) were used to evaluate LDALR. All parameter model tuning and testing, *e.g.*, using cross-validation, were done using only the training dataset *L*. Evaluation, as will be discussed in this section, was done using documents in the dataset *Q*. Documents in *Q *were never used for training and parameter tuning. Furthermore, we have extracted the inclusion and exclusion criteria individually from our data collection. The total number of criteria (inclusive of documents in *L *and *Q*) is 462,459 of which 61% are exclusion criteria and 39% are of the inclusion type. Table 1 shows the details.

**Table 1:** Criteria Counts

	count	pct
inclusion criteria	178,178	39%
exclusion criteria	284,281	61%
Total	462,459	100%

We took advantage of earlier studies, *e.g.*, [12,17-19], suggesting that the use of the PICO [20] framework and its variants, *e.g.*, PIBOSO [21], are helpful in the clinical question-answering problem domain. In this paper, we have only evaluated the use of PICO deferring the investigation of its variants for future work. The PICO acronym stands for: P - patient/problem, I - intervention,exposure,prognostic factor, C - comparison (alternatives considered if any), O - outcome. Instead of using all the words in a document as features we only used words from parts of the document that correspond to PICO, along with the criteria words, thus reducing our dimension. Since the data we downloaded from the website http://www.clinicaltrials.gov are in XML format we took advantage of the tags and extracted PICO data using them. These tags are shown in Table 2.

**Table 2:** XML tags used to extract PICO data

	XML tags
P	brief_summary, detailed_description condition, official_title, brief_title
I	Intervention
C	arm_group
O	primary_outcome, secondary_outcome

## Experiment setup

In our experiments, we applied anchored training as outlined in Algorithm 1. We applied the unsupervised learning method LDA varying the number of topics *n *over the dataset *L*. In order to form the positive instances of topic *i *we used a threshold of *κ*_1 _= 0.02 and to form the negative instances of topic *i *we used a threshold of *κ*_2 _= 0. LDA was applied to a collection of documents in the training set *L *which contain only the PICO portions of the original document. The collection has 39,782 files and has a total of 121,259 words.

Table 3 shows an example of topics and the corresponding words that have the highest probability under these topics. It is important to point out that although LDA can provide clusters of documents that can be labeled under a specific topic, these documents vary in the level to which they belong to that topic, *i.e.*, their corresponding topic proportions could widely differ. For example, in Table 4 the documents NCT00000174e8 and NCT00000408e11 under topic3 have different levels of membership, 0.08 and 0.14, respectively. In fact, the average topic proportion inferred by LDA on the positive examples we used as part of the training sets for pseudo-labels is only 0.08. In contrast, if all positive examples were to belong only to a single topic as in a standard labeled set, this value would be 1.0, so there is a fair amount of noise from the perspective of cluster membership. One of our goals in using LDA is to get a crude approximation of good candidate training examples for an 'anchor'. As we shall see, whether or not something can actually be learned from these noisy examples is related but an entirely different matter. In our implementation the only set of constraints we place on the value of topic proportions are those set by *κ*_1 _and *κ*_2_.

**Table 3:** Illustration of topics (2 out of 5 topics) and corresponding top words

LDA topic #	top words
1	female, study, breast, feeding, subjects potential, contraception, childbearing, drug
3	consent, informed, study, patient subject, inability, comply, give, protocol

**Table 4:** Illustration of topic proportions (5 topics)

document id	topic1	topic2	topic3	topic4	topic5
NCT00000174e8	0.44	0.15	0.08	0.18	0.15
NCT00000408e11	0.86	0.0	0.14	0.0	0.0

As explained above, the first step in anchored training is finding good candidates to be used as training data for a supervised learning method. The data from the PICO portions of the documents are better suited to be used for this initial approximation. This is in contrast to using data from both the PICO portions and criteria, since the latter can be best exploited to guide the parameter fitting in the refinement step. Since our goal is to find the most appropriate eligibility criteria, it is more reasonable to use the criteria data in the second step of the anchored training process to further refine the models and tune them towards finding the most appropriate eligibility criteria.

For the results reported here using the supervised method, we only used unigrams as features. No stemming was applied but common words appearing in a stopword list, punctuations, and digits were eliminated. A training instance is a bag-of-words representation and is a vector of binary features. The feature set of a training instance is a combination of features from a criterion and the PICO features of the same document that same criterion belongs to. Each criterion is mapped to a single training instance. So, if a document has 4 inclusion criteria and 6 exclusion criteria, there is a total of 10 training instances from that document.

To make a decision on which type of supervised learning method to use, we first ran LDA setting the number of topics to *n *= 100. The value of *n *was arbitrarily set but the value is within the range of what others have found useful, *e.g.*, [5,15]. Then we randomly picked 10 topics from out of the 100 topics and evaluated different supervised learning methods and implementations on these 10 topics. We tried several other techniques such as Naive Bayes and SVM as well as other implementations *e.g.*, using WEKA [22]. The LIBLINEAR toolkit gave the fastest and best results. We used *L*_2_-regularized logistic regression from the LIBLINEAR toolkit and built a set of logistic regression models for inclusion and another set of logistic regression models for exclusion criteria. The average number of features in our trained models for number of topics *n *= 100 is around 60,000. The combined total number of unique features for all the inclusion models is 131,772. All the exclusion models have a combined total of 132,506 unique features. Table 5 shows the average number of features and average training size for inclusion and exclusion models.

**Table 5:** Feature and Train Size of Logistic Regression Models

	avg # features	avg train size
inclusion models	60,589	36,505
exclusion models	61,368	58,105

We also ran experiments to determine whether the use of PICO improves accuracy. Results of the experiments indicate that the use of data from portions of a document corresponding to PICO gives a significant boost to a model's discriminative ability. This is supported by the results shown in Table 6 which tabulates the average accuracy (via 10-fold cross validation) over 10 randomly chosen topics using an *L*_2_-regularized logistic regression model. The test data associated with the results in Table 6 have equal number of positive and negative instances. The positive instances for a topic *i *were from documents with topic *i *proportion of at least *κ*_1 _= 0.02. Similarly, the negative instances for a topic *i *were from documents with topic *i *proportion of *κ*_2 _= 0.

**Table 6:** Logistic Regression Average Accuracy (10-fold CV)

	criteria only	criteria+PICO
inclusion models	63.43	79.80
exclusion models	61.95	79.51

Since the number of positive and negative examples are equally distributed, a simple random guess would provide 50% accuracy. Therefore, the results in Table 6 suggest that the use of words from criteria alone improved the models' predictive accuracy by about 25% over a random baseline. If words from criteria are combined with words from PICO, an improvement of about 60% over a random baseline can be achieved. Furthermore, since we were particularly interested in scoring a given set of criteria, *i.e.*, we need to query the model given the criteria, we did not investigate models that used words solely from PICO as features in the logistic regression model because they are not useful for our purpose.

In order to evaluate LDALR, our goal in the experiment is straightforward: *pick a randomly drawn document d_τ _from a previously unseen document collection (dataset Q) and attempt to automatically reconstruct its eligibility criteria*. To help in the reconstruction process, we need some documents *d_j_, d*_j _*≠ d_τ _*from which the components of the target eligibility criteria will be constructed. We can obviously pick random documents from dataset *Q *but a realistic assumption would be that users will pick documents that are related to the target *d_τ_*. Let's call these documents, neighbors of *d_τ_*. We denote a neighbor of *d_τ _*by *d_γ _*and the collection of neighbors by *D_γ _*. We assume that such relatedness is measured by a similarity function and for our experiment we used the cosine similarity since it is one of the most commonly used similarity measures. The cosine similarity between *d_τ _*and *d_γ _*is measured using text from the entire document.

We can now formally state the goal of the experiment. Let *τ *be the set of target criteria, *γ *be the set of candidate criteria, and *s_i _*be a criterion. We want to find the assignment *g *: *γ *→ *τ *such that

(6) $$\underset{s_{i}\in \gamma }{\text{max}}\overset{\left|\tau \right|}{\underset{i=1}{\sum }}\phi \left(g\left(s_{i}\right),s_{i}\right)$$

where *ϕ *can be any similarity function, *e.g.*, cosine similarity.

The evaluation was done in the following manner: A target document *d_τ _*was randomly drawn from the test set *Q*. The components that were used to reconstruct *τ *were formed by collecting all criteria *γ *from each of the neighbors:

$$\gamma =\left\{s|s\;\text{is a criterion of }d_{\gamma }\text{ and }\phi \left(d_{\tau },d_{\gamma }\right)\geq 0.30\right\}$$

In practice, the set *D_γ _*= {*d_γ_*} is decided by the user. *D_γ _*can also be a combination of user-specified documents and documents added by the system automatically (within a threshold) by expanding the original search query used by the user. For this experiment, however, we assumed that *D_γ _*is a set of documents that are similar to the target document *d_τ _*by at least a cosine similarity of 0.30. The similarity value 0.30 was arbitrarily chosen.

It is important to note that the complete recovery of the target *τ *is *only *possible in the case where the same set of criteria can be found in *γ*, either scattered over different documents in *D_γ _*or fully intact in one document *d_γ _*. Since this is not typically the scenario in practice, we compute a *theoretical *limit, *i.e.*, using the set most similar to the target that we can construct from *D_γ _*, assuming the target eligibility criteria τ are completely known. Note that our system does not do this. We only perform this computation separately to evaluate our work. With this computation we can compare what LDALR produces and what, theoretically, can be produced from the given collection regardless of any algorithm.

## Experiment results

**Figure 1:**
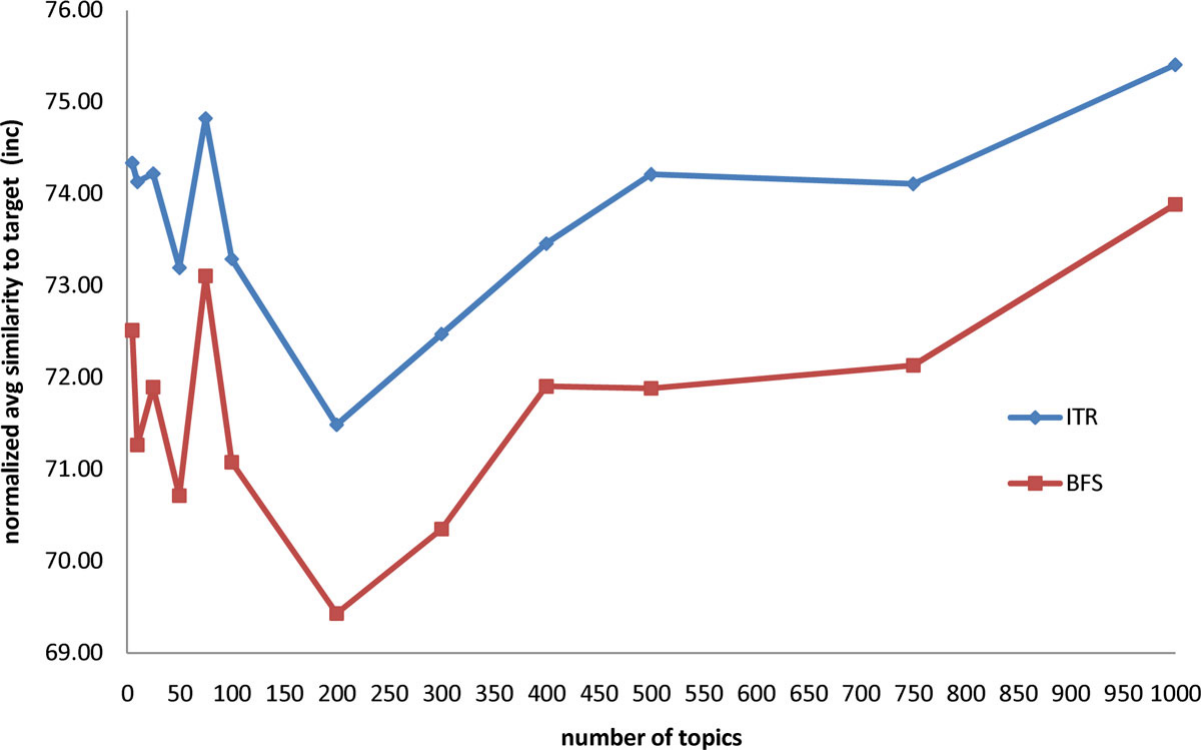
**Normalized average similarity (inclusion) for different number of topics**.

**Figure 2:**
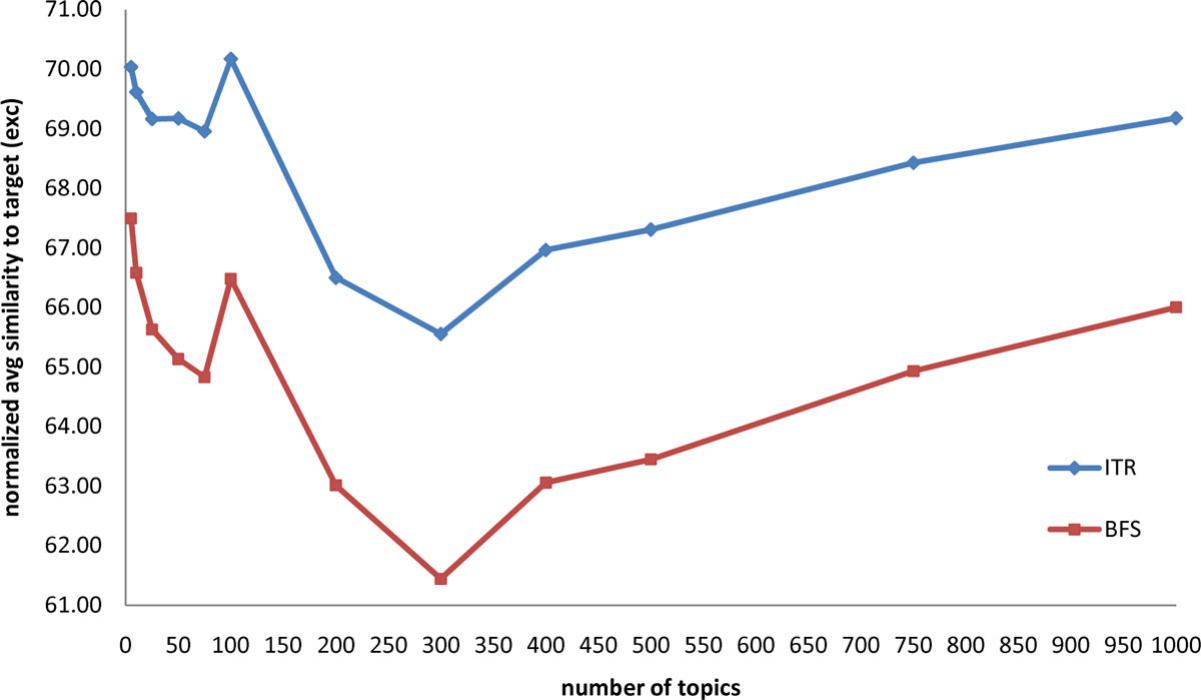
**Normalized average similarity (exclusion) for different number of topics**.

We ran our experiment in a cluster environment using GXP [23], a shell for distributed multi-cluster environment and collected a total of 128 observation points. Each observation point corresponds to a randomly drawn target document. Table 7 gives the average counts of neighbors, criteria (*τ*) and candidates (*γ*). On the average, there are roughly 24 documents that have a cosine similarity of at least 0.3 with the target document. Each randomly drawn target document has on the average, 5 inclusion criteria and 7 exclusion criteria. Note that the proportion of inclusion and exclusion criteria in a given randomly drawn target document is consistent with the proportion shown in Table 1*i.e.*, roughly 40% inclusion criteria and 60% exclusion criteria. As Table 7 shows, for every target inclusion criterion there are, on the average, about 24 candidate criteria that are available as choices and similarly, for every target inclusion criterion about 25 candidate criteria are available as choices. Tables 8 and 9 have the summary of the experiment results for number of topics = 75 and 100, respectively. The respective number of topics correspond to the optimal values for LDALR for the dataset we used (see Figure 1 and 2).

**Table 7:** Average Counts

	neighbors	criteria	candidates
inclusion	23.79	5.03	120.12
exclusion	23.79	6.95	173.12

**Table 8:** Inclusion Criteria Results, Exclusion Criteria Results, Number of Topics = 75

inclusion	LMT	ITR	BFS	RND
avg sim	0.581	0.435	0.425	0.051
normalized sim	100.00	74.823	73.107	8.806
% vs random		849.64	830.16	100.00

**Table 9:** Exclusion Criteria Results, Number of Topics = 100

exclusion	LMT	ITR	BFS	RND
avg sim	0.524	0.368	0.349	0.039
normalized sim	100.00	70.173	66.481	7.358
% vs random		953.76	903.58	100.00

The notations LMT, RND, BFS, and ITR mean the following:

1. Theoretical limit (LMT): Given a set of target criteria *τ *and a set of candidate criteria *γ*, for each *s*_τ _∈ τ, assign a criterion *s_γ _*∈ *γ *such that Eq. (6) is satisfied. In this *O*(|*τ*||*γ*|) scheme, |*τ*| passes are made and for each pass |*γ*| criteria are checked in which the most similar candidate criterion is chosen. Note that this allows a previously chosen *s*_γ _to be chosen again for a different *s_τ_*. The set of candidate criteria *γ *is *not *restricted and includes all possible candidates.

2. Random (RND): Given a set of target criteria *τ *and a set of candidate criteria *γ*, for each *s_τ _*∈ *τ, randomly *assign a criterion *s_γ _*∈ *γ*. *τ *is the set of all criteria collected from *D_τ_*.

3. Best-First Search (BFS): Given a set of target criteria *τ *and a set of candidate criteria *γ*, for each *s_τ _*∈ *τ *formulate the assignment using a best-first search greedy method using the current sum of cosine similarity as a guide to choose which search path to take. The set of candidate criteria *γ *must only be from the top 30% of the ranked criteria. The candidate criteria are ordered using LDALR. The 30% was arbitrarily chosen to match a 30 − 50 set size for display to the user.

4. Iterative(ITR): Given a set of target criteria *τ *and a set of candidate criteria *γ*, for each *s_τ _*∈ *τ*, formulate the assignment by choosing the most similar *s_γ_*. In this *O*(|*τ*||*γ*|) scheme, |*τ*| passes are made and for each pass |*γ*| criteria are checked in which the most similar candidate criterion is chosen. Although slower than BFS, it could potentially find better solutions than BFS. Note that this also allows a previously chosen *s_γ _*to be chosen again for a different *s_τ_*. Although a repair is possible to force the output of ITR to be a solution, we have not implemented such strategy in this experiment. As with BFS, the set of candidate criteria *γ *is restricted to the top 30% of the ranked criteria, ordered by LDALR.

Note that in practice the maximum similarity of a solution would lie between BFS and ITR values. ITR is sometimes not a solution because it is possible that the same candidate criterion will be mapped to two different target criteria. BFS is always a solution but not necessarily optimal.

If the scoring models do not in fact help in finding a solution, then we would expect the performance of BFS and ITR to be no significantly different from the random method RND. The second row of Tables 8 and 9 provide the average similarity values of the solutions to the original target criteria *τ*. In Table 8, LMT has a value of 0.581. This means that given the collection of neighbors *D_γ_*, the average best assignment *g *that satisfies Eq. (6) is only 0.581 similar to the target *τ*. This is in the case (as explained above) where the target *τ *is completely known. In practice, *τ *is unknown.

On the other hand, if one were to formulate the assignment *g *so that the targets *s_τ _*are assigned a random choice of criteria from the entire set *γ *(note that the allowed choices for ITR and BFS are restricted to a smaller set), such method can only achieve a similarity value of 0.051 with the target criteria *τ*. ITR and BFS achieves a similarity of at least 0.42.

Now, if we put this in perspective and assign whatever the LMT value is as 100%, then we have the normalized values which are in the third row of Table 8. According to the normalized values, ITR and BFS can find assignments which are respectively 75% and 73% similar to what is possible. ITR and BFS methods do this with the target criteria *τ completely hidden *from the system. As discussed above, ITR and BFS chose possible assignments from the top of an ordered list produced by our LDALR algorithm. In contrast, a random method could only achieve 9% similarity. The last row of Table 8 indicate that the output of our LDALR algorithm can produce solutions that are about 8 times better than a random method.

Table 9 contains results for the exclusion criteria. The normalized values indicate that ITR and BFS formulate assignments that are respectively 70% and 66% similar to the hidden target criteria while a random method can only achieve 7% similarity. The last row of Table 9 suggests that the ranking provided by our LDALR algorithm can result in solutions that are about 9 times better than a random method.

We also ran experiments to determine whether different values for *n*, the number of topics, affect performance and whether or not there is a particular number of topics where LDALR's performance is at its optimal. In particular, we ran experiments varying the number of topics between 5 and 1000. Results of the experiments are shown in Figure 1 and 2.

Figure 1 shows how the normalized average similarity values vary with respect to different values of the number of topics for inclusion models. Both the ITR and BFS average similarity value peaks at number of topics equal to 75 and proceeds to drop to its lowest point at number of topics equal to 200 before steadily climbing back up again. A similar behavior can be observed for exclusion models in Figure 2 except that for exclusion models the peak is at 100 and the lowest point is at 300. Both graphs suggest that model overfitting starts to happen at some specific value of the number of topics. Overfitting [24,25] occurs when the model becomes excessively complex. In this case when the number of topics becomes too large, the model captures the peculiarity of the training data and will likely not generalize well on new data. The increasing average similarity values after number of topics equal to 200 for inclusion models (respectively, after number of topics equal to 300 for exclusion models) suggests overfitting. To avoid overfitting, care needs to be taken so that the number of topics used is not too large. The results plotted in Figure 1 and 2 suggest that with our dataset, the optimal choice for the number of topics for the inclusion models (resp. exclusion models) is around 75 (resp. 100).

## Eligibility criteria comparison tool

As part of our plan to conduct more user study and testing, we have developed an eligibility criteria comparison tool. The tool is intended to facilitate the elicitation of user-provided eligibility criteria rank ordering by allowing a domain expert or clinician to easily view results from different methods. It is accessible online via the link: http://nactem.ac.uk/EligibilityComparison. To our knowledge, no publicly accessible tool exists other than what we have described in this section, that facilitates the comparison of outputs that are rank-ordered using different methods. We have developed this tool to help better measure the usability and effectiveness of eligibility criteria recommended by recent methods, LDALR [1] and ASCOT [6], and any future methods of assessing the appropriateness of eligibility criteria in clinical trial protocols.

More specifically, we implemented a web-service that allows a user to compare the eligibility criteria recommendation of LDALR and ASCOT. The online comparison tool can be used in conjunction with an existing clinical trial protocols search interface that allows a user to find and search for relevant documents. The latter is accessible via the link: http://www.nactem.ac.uk/ClinicalTrialProtocols. As soon as relevant documents are identified and selected by the user through a search, the eligibility criteria comparison tool takes as input the collection of eligibility criteria associated with these documents, and invokes the two aforementioned eligibility criteria recommendation engines. It then presents the outputs to the user for comparison.

**Figure 3:**
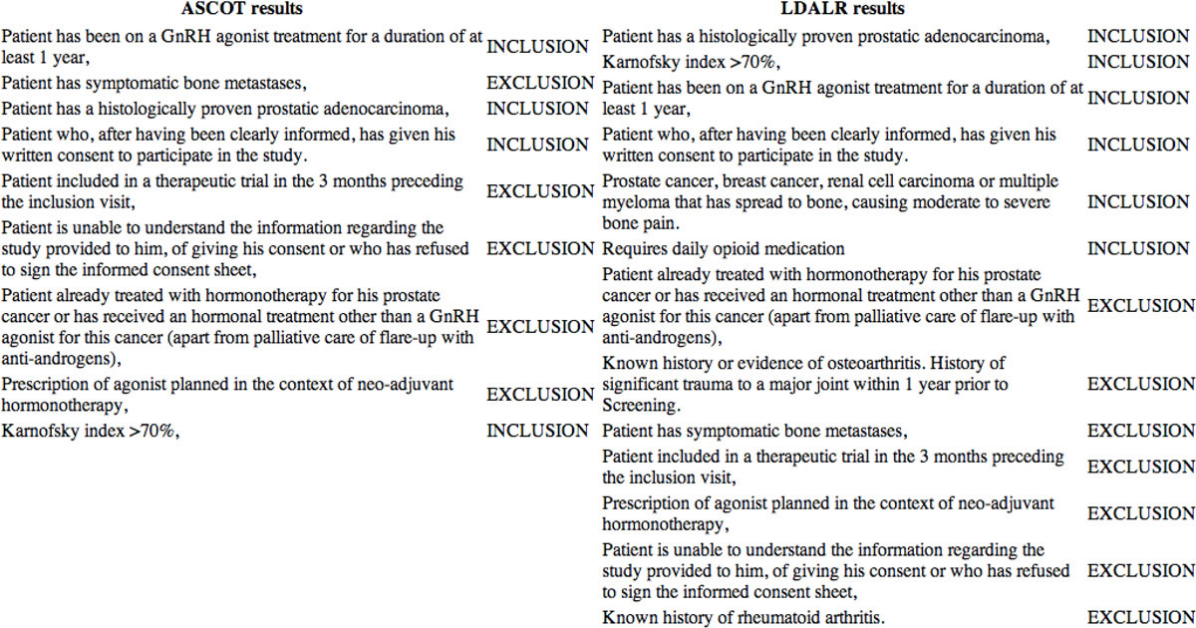
**Eligibility Criteria Comparison Tool Snaphot**.

Figure 3 shows an example of the output of the eligibility criteria comparison tool. For illustration purposes, we only show the output of two documents. The comparison tool was run using a collection of eligibility criteria from two documents: the clinical trial protocols NCT00545129 and NCT01011751. These two documents belong to the set of documents returned after using the search query: "prostate cancer" AND "bone metastases" AND "palliative care". ASCOT returns less criteria than LDALR since the former does not retrieve relevant criteria originating from documents outside of the query results. In addition, ASCOT presents results in the order of importance irrespective of the criterion type, *i.e.*, irrespective of whether it is an inclusion or exclusion criterion. The current implementation of LDALR presents the ordered criteria readily grouped according to criterion type. However, since a score is associated with each eligibility criterion it is also possible to present the criteria order using the score, irrespective of the criterion type.

## Conclusions

We have presented a general framework for building machine learning models *without *labeled data. In this work, we have demonstrated how such framework can be applied to the clinical trials domain. More particularly, we have used a specific instance of the framework, LDALR, to discover and infer appropriate eligibility criteria in clinical trial protocols. The results of our experiments suggest that even without labeled data, it is possible to build useful machine learning models and that leveraging these models could result in significant gains (at least 8 times better) over a random method.

We believe that our results could be further improved with the use of domain-specific knowledge, *e.g.*, UMLS SNOMED CT terms and ontology, as well as annotations, such as ERGO [26] which can be used as features for machine learning models. Hence, as part of our future plan we will conduct more experiments using data enhanced with annotations and domain-specific knowledge. Moreover, the use of n-grams and possibly a small library of templates could be further explored to leverage context as well as detect common patterns such as those typically used with age criteria.

In addition, we plan to conduct user study and testing to better measure the usability and effectiveness of the recommended eligibility criteria. To help us and the broader research community in the assessment and evaluation, we have developed the eligibility criteria comparison tool which we have described in this paper. Our goal is to better assess the eligibility criteria selected, missed, or erroneously included by LDALR, and to compare them with the results of other methods using data from user study and testing. By making the online comparison tool accessible to the research community, we hope to promote deeper interest and collaboration among researchers by providing a practical tool to evaluate and compare alternative methods for finding appropriate eligibility criteria in clinical trial protocols.

## Competing interests

The authors declare that they have no competing interests.

## Authors' contributions

AR formulated the LDALR algorithm, implemented it, designed and conducted the experiments, and drafted all sections of the manuscript except for the section on eligibility criteria comparison tool. IK was responsible for putting together the comparison tool and also drafted the section discussing the tool. AS supervised the work. All authors have read and approved the final version of the manuscript.

## Authors' information

The work reported in this paper, with the exception of the eligibility criteria comparison tool, was conducted while AR was a Research Associate at The University of Manchester. He is currently affiliated with eBay Inc., 2065 Hamilton Avenue San Jose, California 95125, USA. Currently, IK is a Research Staff in the School of Computer Science at the University of Manchester and the National Center for Text Mining. AS is a Professor in the School of Computer Science at the University of Manchester and the Director of the National Center for Text Mining.

## Declarations

The publication costs for this article were funded by grants from JISC, Digging into Data, and Integrated Social History Environment (ISHER).

This article has been published as part of *BMC Medical Informatics and Decision Making *Volume 13 Supplement 1, 2013: Proceedings of the ACM Sixth International Workshop on Data and Text Mining in Biomedical Informatics (DTMBio 2012). The full contents of the supplement are available online at http://www.biomedcentral.com/bmcmedinformdecismak/supplements/13/S1.
